# Characteristics of Red Deer (*Cervus elaphus*) Milk: Lactational Changes in Composition and Processing Impacts on Structural and Gelation Properties

**DOI:** 10.3390/foods12071517

**Published:** 2023-04-03

**Authors:** Siqi Li, Ashish Saharawat, Aiqian Ye, Anant Dave, Harjinder Singh

**Affiliations:** Riddet Institute, Massey University, Private Bag 11 222, Palmerston North 4442, New Zealanda.m.ye@massey.ac.nz (A.Y.);

**Keywords:** deer milk, lactation, composition, fatty acids, milk proteins, milk structures, milk gels

## Abstract

In an increasingly diversified global market, milk of minor dairy species has gained interest as a novel and premium source of nutrition. Relative to the major dairy species, much is lacking in our understanding of red deer (*Cervus elaphus*) milk. In this study, we characterized the compositions (macronutrients, minerals, fatty acids, and proteins) of red deer milk and their variations throughout lactation. We also investigated the structures, physical properties, and gelation (acid- and rennet-induced) properties of deer milk and how they are impacted by typical processing treatments (e.g., homogenization and pasteurization). We identified unique features in the composition of deer milk, including being richer in protein, fat, calcium, zinc, iodine, branched-chain fatty acids, and α-linolenic acid than other ruminant milks. Different deer milk components displayed diverse variation patterns over the lactation cycle, many of which were different from those demonstrated in other ruminant species. Other physicochemical features of deer milk were identified, such as its markedly larger fat globules. Processing treatments were demonstrated to alter the structural and gelation properties of deer milk. Most of the gelation properties of deer milk resembled that of bovine milk more than ovine and caprine milks. This study furthers our understanding of red deer milk and will aid in its processing and applications in novel products.

## 1. Introduction

Red deer (*Cervus elaphus*) are domesticated primarily for meat, velvet, leather, and hunting purposes [1,2]. New Zealand is one of the leading countries in deer farming. In addition to the main exports of venison and velvet, the New Zealand deer industry has been exploring red deer milk for its value as a novel ingredient in food, nutritional, and cosmetic products [1,2]. Unlike other common dairy ruminant species such as cattle, goats, and sheep, which belong to the family Bovidae, deer belong to the family Cervidae. Possibly arising from this genetic difference, red deer milk has some unique characteristics, such as being naturally nutrient dense with markedly higher contents of protein, fat, and calcium compared with other ruminants’ milk [3]. Moreover, the lactation cycle of red deer is shorter than those of other common ruminant species and typically lasts for 16–20 weeks [2,4,5]. Great potential lies in utilizing the unique characteristics of red deer milk in novel products, and a better understanding of its production, natural variation, and functionalities will lay the foundation for future applications.

To date, research on deer milk has focused mainly on its compositional features [1,5,6,7,8]. A few studies have investigated some of the physicochemical properties such as ethanol stability [9], gelation properties [2,10], and protein characteristics [11,12] of deer milk. It is well known that the compositional and physicochemical properties of milk vary over the lactation cycle; these properties could not only determine the nutritional quality of the milk, but also influence its techno-functional properties [13,14,15]. For example, late-lactation bovine milk was reported to have a long clotting time and low curd firmness during cheesemaking [16], whereas acid-induced gels made from late-lactation sheep milk were reported to have the lowest gel strength over the lactation cycle [17]. In addition to the natural variations, milk components are organized in unique structures that are maintained dynamically by complex physicochemical interactions, which are altered under different processing treatments such as homogenization, heating, and acidification [18]. A better understanding of deer milk properties, their variations over the lactation cycle, and processing-induced changes will enable the applications of deer milk as a novel premium food to be diversified.

In this work, we studied the compositions (macronutrients, minerals, fatty acids, and proteins) of New Zealand red deer milk throughout the lactation cycle and characterized the physicochemical features and gelation properties (acid- and rennet-induced) of differently processed deer milks. Similar methods had been used for previous studies on bovine, ovine, and caprine milks produced in New Zealand [14,19], allowing identification of the similarities and differences in ruminant milk characteristics and enabling optimized applications.

## 2. Materials and Methods

### 2.1. Milk Sampling

Raw deer milk was collected from a Pāmu Deer Milk supply partner farm in Gore, New Zealand. The herd consisted of 120 lactating does. The deer milk was collected on eight different occasions, mostly at fortnightly intervals, from the 3rd to the 16th week of lactation, i.e., from November 2020 to February 2021. Throughout the milking season, the does were mostly pasture fed while also provided a grain-based supplement. There was no systematic change in the diet over the season.

Aliquots of all milks were stored at −80 °C and were used to study the impact of lactation on the milk composition (proximate composition, minerals, proteins, and fatty acids). Milks collected in January and February 2021 were also studied for their processing properties and acid- and rennet-induced gelation properties (*n* = 3–4). The processing of the milks was carried out at the FoodPilot of Massey University (Palmerston North, New Zealand). Three different processing conditions were studied: (1) pasteurization (75 °C/15 s); (2) two-stage homogenization and pasteurization (20 and 5 MPa; 75 °C/15 s); (3) two-stage homogenization (20 and 5 MPa) and heating at 95 °C for 5 min. Part of the fresh milk was skimmed by centrifugation (3000× *g* for 15 min) and heated (75 °C/15 s and 95 °C for 5 min) for further analyses (casein micelle size, ionic calcium, and protein composition). Liquid milk samples were preserved with sodium azide (0.02% *w*/*w*) and were stored at 4 °C before analysis.

### 2.2. Compositional Analyses

The impacts of lactation on different compositions of the red deer milk, including proximate composition (total solids, fat, protein, lactose, and casein), mineral composition (calcium, magnesium, potassium, sodium, phosphorus, chloride, copper, iodine, selenium, and zinc), protein composition, and fatty acid composition were analyzed using methods described in Li et al. [19].

Briefly, the proximate composition was determined on a MilkoScan FT1 (Foss Electric, Hillerød, Denmark). To fit the calibration range of the device, the deer milk samples were mixed 1:1 (wt/wt) with reverse osmosis water prior to the measurement.

The mineral composition was determined using inductively coupled plasma–optical emission spectrometry (calcium, magnesium, potassium, sodium, and phosphorus), inductively coupled plasma–mass spectrometry (copper, iodine, selenium, and zinc), and potentiometric titration (chloride, AOAC 971.27). The soluble fractions of calcium, magnesium, and phosphorus in the deer milk were determined by measuring the supernatant following ultracentrifugation at 63,000× *g* for 60 min at 20 °C.

The fatty acid composition of the deer milk was analyzed using gas chromatography with flame ionization detection (Shimadzu Nexis GC-2030, Shimadzu, Kyoto, Japan) on a Restek Rx 2330 column (105 m × 0.25 mm ID, 0.20 μm film thickness) as described in Li et al. [19].

The protein composition of the deer milk was analyzed using both sodium dodecyl sulfate polyacrylamide gel electrophoresis (SDS-PAGE) and high-performance liquid chromatography (HPLC). SDS-PAGE was performed as described by Ye et al. [20]. Deer skim milk was diluted in the sample buffer to reach a final concentration of approximately 1 mg/mL. Precision Plus Protein Dual Xtra Prestained Protein Standard (2–250 kDa, Bio-Rad Laboratories, Hercules, CA, USA) was loaded with the milk sample as a molecular weight marker. The SDS-PAGE gel was scanned using the Gel Doc XR+ system and analyzed for band staining intensity using Image Lab 5.2.1 software (Bio-Rad Laboratories, Hercules, CA, USA). The deer skim milk was also analyzed for protein composition using an HPLC method previously applied to other ruminant milks, as described by Li et al. [19]. To verify whey protein peaks in the chromatogram, whey proteins were separated using acid precipitation (to pH 4.6), as modified from Vasbinder and De Kruif [21]. A total of 0.4 mL of deer skim milk was well mixed with 780 μL of water and 60 μL of 10% acetic acid, left to stand for 10 min, and then mixed with 60 μL of 1M sodium acetate and 700 μL of water. This mixture was kept for 60 min at room temperature before centrifugation at 3000× *g* for 5 min. The supernatant containing whey proteins was sampled and subjected to analysis by HPLC.

### 2.3. Physicochemical Properties

The physicochemical properties of raw deer milk were characterized as described previously [14,19]. The fat globule size was determined using a Malvern MasterSizer 2000 (Malvern Instruments Ltd., Malvern, UK) after chemically dissociating the casein micelles using a solution containing 50 mM EDTA and 2% (wt/wt) SDS. The casein micelle size was determined in raw and heated skim milks with a Malvern Zetasizer Nano ZS after 100 times dilution with an imidazole buffer [22]. Ethanol stability was determined as the highest ethanol concentration that did not induce coagulation of raw deer skim milk. The ionic calcium concentration was measured with a calcium-selective electrode (Orion 9720BNWP; Thermo Fisher Scientific, Waltham, MA, USA), as described previously [14]. The viscosity (mPa·s) of the deer milk was measured using an AR-G2 magnetic bearing rheometer (TA Instruments, Crawley, UK) paired with a concentric cylinder geometry. It was measured during a shear rate sweep from 0.01 to 1000 s^–1^ at 20 °C (over 3 min) and the viscosity measured at the shear rate of 100 s^–1^ is reported.

### 2.4. Gelation Properties

The acid- and rennet-induced gelation properties of raw, pasteurized, and homogenized and pasteurized deer milks were studied using the AR-G2 rheometer (TA Instruments), as described previously [17].

For acid-induced gelation, the milk sample was acidified using 3% (wt/wt) glucono-δ-lactone (GDL) at 30 °C and stirred for 2 min. Then a 20 mL portion of the acidified milk was transferred to the rheometer for a small amplitude oscillatory test for 8 h at 30 °C (frequency of 0.1 Hz, strain of 1%). Simultaneous to the rheological measurement, the pH of the rest of the acidified milk was recorded using an HI-2202 Edge^®^blu pH meter (Hanna Instruments, Woonsocket, RI, USA) in a temperature-controlled jacket vessel at 30 °C. The concentration of GDL used for the deer milk was selected based on preliminary trials to achieve an approximate pH of 4.2 after 8 h of incubation. The acid gelation time was defined as the time from GDL addition to the moment when the storage modulus (G′) was greater than 1.0 Pa, when the pH was recorded as the acid gelation pH. After the 8-h incubation period, the final G′ and the tan δ (the loss modulus/storage modulus ratio) were recorded.

The rennet-induced gelation was induced using chymosin (HANNILASE^®^ XP 1050 NB; Christian Hansen A/S, Horsholm, Denmark) at a concentration of 38 international milk clotting units (IMCU)/L of milk. The milk was adjusted to pH 6.5 with 0.5 M HCl before rennet addition. The oscillation test was conducted at 32 °C for 60 min (frequency of 0.1 Hz, strain of 1%). The gelation time from coagulant addition, and the final G′ and tan δ values were recorded.

### 2.5. Statistical Analysis

All analyses were at least duplicated. Statistical analysis was conducted using Minitab 19. Means, standard deviations, and coefficients of variation (CV) were determined. Error bars in the figures indicate standard deviations. Variations in some compositional parameters over the lactation (from week 3 to week 16) were fitted to linear, quadratic, and cubic regression models to indicate the patterns of variation. Significant differences between groups (e.g., processing treatments) were analyzed using one-way analysis of variance (ANOVA) followed by the Tukey post hoc test. Significant correlations and Pearson correlation coefficients (*r*) between parameters were also determined.

## 3. Results and Discussion

### 3.1. Deer Milk Compositions over Different Stages of Lactation

#### 3.1.1. Proximate and Mineral Compositions

**Figure 1:**
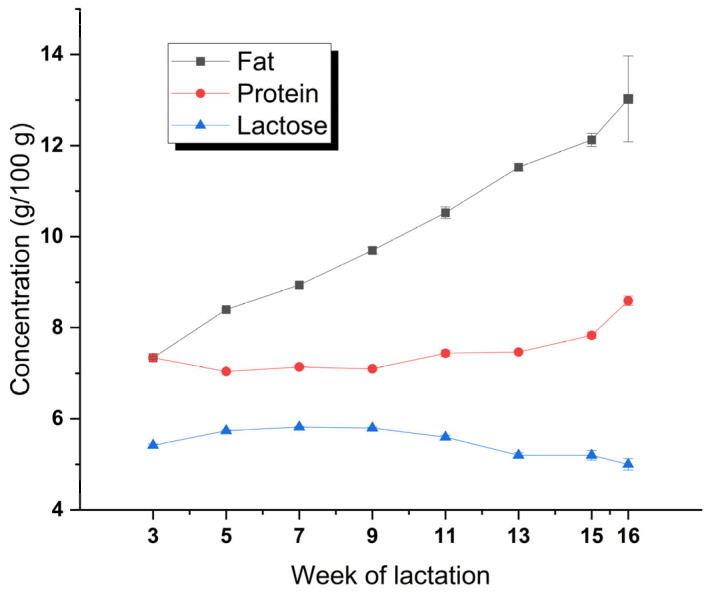
Variations in the fat, protein, and lactose contents of deer milk over the lactation.

The proximate composition of the deer milk throughout the lactation is presented in Figure 1. The mean contents of fat, protein, lactose, and total solids of the deer milk were 10.2 ± 0.7, 7.5 ± 0.2, 5.5 ± 0.1, and 23.7 ± 0.8 g/100 g, respectively. The percentage of casein in total protein was determined as 81.7 ± 1.1%, which was fairly stable over the lactation. Over the lactation cycle, the contents of both fat and protein increased, whereas the lactose content decreased. The increase in fat content was greater than the increase in protein content, being from 7.3% in week 3, to 13.0% in week 16, and increased in a linear fashion (linear regression *R*^2^ = 0.99, *p* < 0.001). The variation in fat content drove the increase in the total solids content over the lactation cycle. The variation in protein content was smaller, ranging from 7.0 to 7.5% in the first 13 weeks, and increased only to 7.8 and 8.6% in the last 2 weeks of lactation. The lactose content displayed the opposite trend to the protein content (*r* = −0.87, *p* < 0.01).

The proximate compositions of the deer milk and their lactational trends were mostly consistent with those reported previously [1,2,5,6,8], although the lactose content of the deer milk in the present study was at the higher end of the reported range. Increases in protein and fat contents and decreases in lactose content towards the end of lactation have also been reported for the milks of other ruminants (cow, sheep, and goat) [14,19,23,24].

The macromineral concentrations in the deer milk over the lactation are shown in Table 1. The main minerals in the deer milk were determined to be calcium (298 ± 18 mg/100 g), phosphorus (208 ± 14 mg/100 g), potassium (136 ± 11 mg/100 g), chloride (77 ± 11 mg/100 g), sodium (38 ± 4 mg/100 g), and magnesium (17 ± 1 mg/100 g). The results were in line with those reviewed by Wang et al. [1]. Interestingly, the calcium concentration of the deer milk in the present study was close to that previously reported in New Zealand [1], and was higher than those reported for the Iberian and Scottish breeds of red deer, which ranged from approximately 160 to 240 mg/100 g [4,7]. The calcium concentration of around 300 mg/100 g is considerably higher than those of other ruminant milks (bovine, ovine, and caprine) [1,19,25].

Over the lactation, the sodium and chloride concentrations increased significantly, whereas the potassium concentration decreased (*p* < 0.01). There was no significant effect of the lactation stage on the calcium, phosphorus, and magnesium concentrations, all of which had limited variation over the lactation, as indicated by their small CV values of 5.8–6.5%. The calcium/phosphorus ratio was 1.44 ± 0.15, with no significant trend over the lactation cycle. Some previous studies reported similar patterns for sodium and potassium over the lactation [4,7], whereas Malacarne et al. [5] did not report a clear lactational trend in these minerals. The increase in sodium and chloride concentrations and decrease in potassium concentration over lactation has also been reported for other ruminant milks [13,19,26]. The rather stable concentrations of calcium, phosphorus, and magnesium over the lactation agreed with results reported previously [4,27]. In addition, these authors noted a trend of decreasing calcium concentration towards the end of the lactation, which also agreed with our finding that the lowest calcium concentration was found in week 16. Vergara et al. [7] reported that the calcium concentration in Iberian red deer milk was stable during lactation but that a pronounced decreasing trend was found in Scottish red deer milk. The stable or decreasing calcium concentration in red deer milk in late lactation as the protein content increased is different from results found for bovine milk, where both the calcium concentration and the protein content increased in late lactation [14,15].

For the milk collected from week 11 to week 16, the soluble fractions of calcium, magnesium, and phosphorus were 21.4 ± 2.9, 57.2 ± 2.1, and 33.2 ± 1.4% (wt/wt) of the total, respectively. The results were similar to those reported for red deer milk by Malacarne et al. [5] (26.9, 56.9, and 30.0%, respectively). The proportions of these soluble minerals were similar to those reported for ovine milk but lower than those reported for bovine and caprine milks [19,26].

The microminerals in the deer milk were also studied over the lactation (Table 1). The mean concentrations were 9.56 ± 1.16, 0.69 ± 0.37, 0.16 ± 0.15, and 0.019 ± 0.013 mg/kg for zinc, iodine, copper, and selenium, respectively. Consistent with previous studies, the red deer milk was markedly more rich than other ruminant milks in zinc [1,19,25]. The iodine concentration was also higher than the concentrations reported for bovine, ovine, and caprine milks, and was around 0.20 mg/kg [25].

Over the lactation, the zinc concentration was quite stable (CV 12%), except for a high level at the beginning (week 3). The other microminerals varied greatly over the lactation, with CV values ranging from 54 to 97%. The selenium concentration was low at around 0.01 mg/kg in the first 11 weeks and increased to 0.02–0.04 mg/kg in weeks 13–16. The iodine concentration fluctuated, with no clear trend. The copper concentration in the deer milk had the most pronounced lactational pattern, being highest during early lactation, decreasing rapidly from 3 to 9 weeks of lactation, and remaining at low levels towards the end.

#### 3.1.2. Fatty Acid Composition

The fatty acid composition of the red deer milk over the lactation is presented in Table 2.

Compared with other ruminant milks, the fatty acid composition of deer milk had some unique features, including an abundance of C4:0, C18:0, branched-chain fatty acids (BCFAs), and some polyunsaturated fatty acids. The most abundant fatty acids in the deer milk were C16:0, C18:0, C18:1 c9, C14:0, and C4:0. The level of C16:0 in the deer milk fat was similar to those in caprine and ovine milks and lower than that in bovine milk, previously reported in New Zealand [19,28,29]. The deer milk fat was more abundant in C18:0 and C4:0 fatty acids and contained less C8–C12 fatty acids, C17:1, and cis-9,trans-11 conjugated linoleic acid (CLA) than bovine, ovine, and caprine milk fats [19,25,29,30]. In addition, the deer milk fat was richer in most BCFAs and some odd-chain fatty acids. Compared with the ovine and caprine milk fats analyzed using the same method and reported in our earlier study [19], the deer milk fat was significantly higher in iso C14, iso C15, anteiso C15, iso C16, iso C17, anteiso C17, and C17:0 (*p* < 0.05), most of which were also higher than results reported for bovine milk [30,31]. The sum of the six BCFAs identified in the present study made up 2.85% of the total fatty acids in the deer milk, i.e., considerably higher than previously reported for bovine milk (1.7–1.8%), ovine milk (1.64%), and caprine milk (1.27%) [19,30,31], although higher BCFA levels have also been reported for these species [32]. The BCFAs and odd-chain fatty acids in ruminant milks are largely derived from rumen bacteria [31,32]. Studies on BCFAs have indicated their potential benefits for human health and development, including anti-inflammatory and anti-cancer activities [32,33]. Their abundance in deer milk compared with other ruminant milks might arise from the diversity of rumen microbiota in different ruminant species. Finally, α-linolenic acid (C18:3 n3) was more abundant in the deer milk fat than in bovine, ovine, and caprine milk fats [19,25,29,30], largely agreeing with the results reported by Lagutin et al. [28].

**Figure 2:**
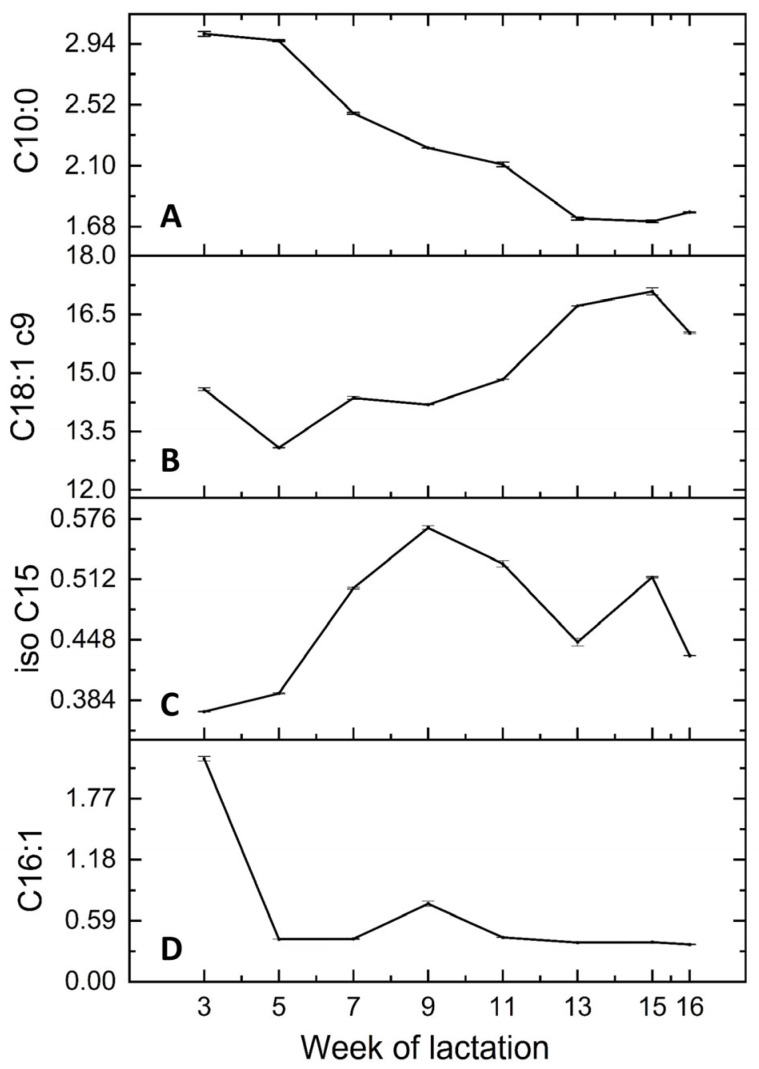
Variation patterns of representative fatty acids in deer milk (g fatty acid/100 g) over the lactation: (**A**) C10:0; (**B**) C18:1 c9; (**C**) iso C15; (**D**) C16:1.

The variations in the individual fatty acids in the deer milk over the lactation are presented in Table 2. Several lactational variation patterns were identified for different groups of fatty acids, and those of representative fatty acids are presented in Figure 2.

1. C6–C14 fatty acids (excluding iso C14) decreased over the lactation (Figure 2A);
2. The abundant long-chain fatty acids, C18:0 and C18:1 c9, increased over the lactation (Figure 2B);
3. All the BCFAs (excluding iso C17) displayed significant quadratic patterns; they were lowest at the beginning of lactation, increased to their highest levels in week 9, and then decreased slightly towards the end of lactation (Figure 2C). Similarly, the odd-chain fatty acids C15:0 and C17:0 increased from week 3 to week 9 but did not decrease as markedly thereafter;
4. The monounsaturated fatty acids C14:1, C16:1, and C18:1 c11 remained stable except for remarkably higher levels in week 3, which were 2 to 4 times higher than the levels in later lactation (Figure 2D);
5. The abundant fatty acids C16:0 and C4:0 had random variation;
6. C18:1 t11 and some polyunsaturated fatty acids, including C18:3 n3 and CLA, fluctuated in cubic patterns. C18:1 t11 and CLA fluctuated in similar patterns over the lactation: decrease–increase–decrease.

The patterns of variation of the deer milk fatty acids over the lactation differed markedly from those that are well established for bovine milk, such as the decrease in C4:0 from early lactation and the highest C16:0 and lowest C18:1 c9 levels in mid-lactation [13,29,34], indicating different physiological evolution and metabolic processes from those of lactating cows. The simultaneous decreases in short- to medium-chain fatty acids and increases in the abundant C18 fatty acids suggest a considerable shift in triacylglycerol composition, which is yet to be investigated. The consistent variation patterns of most of the BCFAs probably arose from their rumen bacterial origin, as mentioned earlier.

Stearoyl-CoA desaturase (SCD) is responsible for the production of some monounsaturated fatty acids and CLA in ruminant milks [19,35]; its activity is indicated by the ratio between the product fatty acid and the substrate fatty acid, as presented in Table 2. The role of SCD could explain the similar variation patterns of C18:1 t11 and CLA, being the substrate and product fatty acids of SCD (Figure 2). Interestingly, the C14, C16, and C18 desaturation ratios all displayed lactation patterns of being highest in week 3, similar to those of C14:1, C16:1, and C18:1 c11. These findings suggest that the transition from colostrum to mature milk in week 3 probably caused the high levels of these fatty acids, in which higher SCD activity might have played a part.

#### 3.1.3. Protein Composition

**Figure 3:**
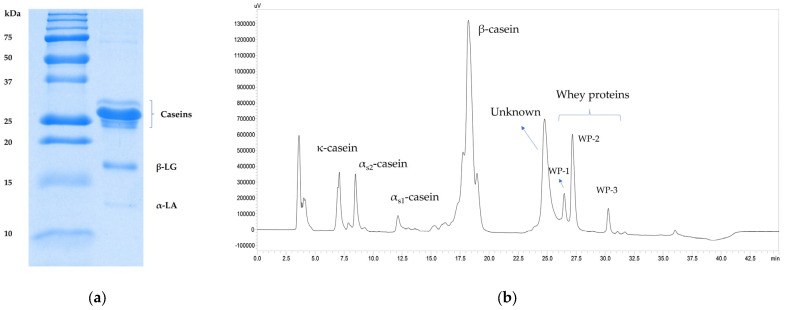
(**a**) SDS-PAGE profile of deer milk proteins (Lane 1, molecular weight marker; Lane 2, deer skim milk; β-LG, β-lactoglobulin; α-LA, α-lactalbumin). (**b**) HPLC profile of deer milk proteins (WP, whey protein).

The protein profiles of the deer milk were analyzed using both SDS-PAGE and HPLC. The SDS-PAGE profile of the deer milk (Figure 3a) largely resembled those of other ruminant milks. Four casein bands of around 25 kDa were separated, with the most abundant casein band making up roughly 50% of the total staining intensity of the lane. Two prominent whey protein bands were identified as β-lactoglobulin and α-lactalbumin, as previously reported [12]. The casein/total protein ratio estimated based on the staining intensity of the bands was 81.4%, in line with that determined using the MilkosScan (Section 3.1.1).

A representative HPLC chromatogram profile is shown in Figure 3b. The caseins were identified based on comparisons with the HPLC profiles of other ruminant milks that had been analyzed using similar methods [14,19]. The most abundant protein in the deer milk was identified to be β-casein, in agreement with results reported by some researchers [1,27]; however, Ha et al. [11] identified the most abundant protein in deer milk as being α_s1_-casein. In the present study, this protein made up approximately 50% of the total peak area in the chromatogram, similar to the staining intensity of the most prominent casein band in the SDS-PAGE profile. Whey protein peaks in the deer milk were verified by analyzing the proteins that were soluble in the milk serum following acid precipitation of caseins at pH 4.6. Three whey protein peaks (WP-1, WP-2 and WP-3, Figure 3b) were verified as acid-soluble, whereas the peak that eluted between the caseins and the whey proteins was labeled as unknown. Analysis of this peak in the skim milk and the milk serum showed that it contained a casein-like protein (acid-insoluble) coeluting with a whey protein (acid-soluble, roughly 10–15% of the peak area of the unknown peak). Together with the three whey protein peaks that eluted later, the whey proteins in the deer milk were separated into four different fractions under the HPLC conditions used in the present study, unlike the two bands in the SDS-PAGE profile (Figure 3a). The whey protein peaks that were separated by HPLC could have contained different genetic variants of whey proteins, as with bovine β-lactoglobulin A and B [36], and other whey proteins such as serum albumin, lactoferrin, and immunoglobulin.

The changes in the protein composition of the deer milk at different stages of lactation were determined using HPLC. Different patterns of variation of the individual proteins were found (Table 3). The proportions of κ-casein (*p* < 0.001) and the unknown protein (*p* < 0.05) increased over the lactation, whereas those of α_s2_-casein and the three whey proteins decreased (*p* < 0.01). The most abundant protein, which was identified to be β-casein, remained stable over the lactation.

To our knowledge, there is no previously published information on the lactational variation of individual deer milk proteins. A few studies have reported that the trend in the casein/protein ratio over the lactation increases [2], remains rather stable [5], or varies depending on the individual doe [37]. In other ruminant milks, the proportion of total whey proteins tends to increase in late lactation, driven by β-lactoglobulin, whereas α-lactalbumin displays a decreasing trend [14,15,19]. It was interesting in the current study to find that all three whey proteins in the deer milk decreased proportionally over the lactation. Moreso, given the stable-to-decreasing trend of the calcium concentration (Table 1), and despite the increase in protein content during late lactation, the physiological processes that are involved in deer milk protein production might be quite different from those of dairy cows and other ruminants.

### 3.2. Physicochemical Properties of Deer Milk

Deer milk samples collected from week 11 to week 16 were studied with respect to the effects of processing on their physicochemical and gelation properties. The physicochemical properties of the differently processed deer milks are shown in Table 4. The mean pH of the deer milk was 6.67; it was unaffected by the processing conditions. The mean ionic calcium concentration of the raw deer milk was 3.34 ± 0.06 mM, higher than those previously reported for New Zealand bovine and ovine milks and similar to that of caprine milk, measured using the same method [14,19]. However, as the total calcium concentration in the deer milk (298 mg/100 g or 74.5 mM) was markedly higher than those in other ruminant milks (approximately 120–200 mg/100 g), the proportion of ionic calcium in the total calcium was only 4.5%, i.e., lower than the proportions reported for caprine, bovine, and ovine milks (11, 8, and 5%, respectively).

All three processing treatments reduced the ionic calcium concentration in the deer milk similarly, to around 3.10 mM (*p* < 0.05). Heating is known to reduce the ionic calcium concentration because of the reduced solubility of calcium phosphates at elevated temperatures, the extent of which typically increases with increasing heating intensity [38]. The significant reduction in the ionic calcium concentration in the deer milk after the rather mild pasteurization, which did not reduce further at greater heating intensity, was interesting. The naturally small fraction of ionic calcium in the deer milk may have played a role.

The mean ethanol stability of the raw deer milk was 59.4 ± 1.3% (Table 4), which was higher than those for caprine and ovine milks (around 50%) but lower than that for bovine milk (around 75%) in New Zealand [14,19]. Similarly, previous studies in Spain reported that the ethanol stability of deer milk was around 65%, higher than that of caprine milk (50%), similar to that of ovine milk (63%), and lower than that of bovine milk [2,9]. Higher concentrations of protein and ionic calcium in bovine milk have been associated with lower ethanol stability because they promote aggregation of the casein micelles [14,39], which may have partially accounted for the lower ethanol stability of the deer milk compared with bovine milk.

The average diameter of the fat globules in the raw deer milk was 6.66 ± 0.10 μm. In agreement with previous studies, the fat globule size of the deer milk was considerably larger than those of bovine milk (4.3–5.4 μm), caprine milk (4.0 μm), and ovine milk (4.5 μm) [2,10,14,19]. The large fat globule size of deer milk has been associated with its high fat content and the relative shortage of fat globule membrane materials [2]. The same mechanism could explain the larger fat globule size during the period of high fat yield during the lactation of dairy cows [14,40]. In addition, the larger fat globule size of the deer milk could also be associated with its fatty acid composition (Table 2). In bovine milk, larger fat globules have been reported to contain more C18:0 and less C12:0 [29,41,42,43], both of which were features of the deer milk fatty acids, compared with other ruminant milks with smaller fat globule sizes.

The average casein micelle size in the raw deer milk was 195 ± 4 nm, which remained similar following pasteurization (199 ± 5 nm) (Table 4). The casein micelle size of the raw deer milk agreed with that reported by Roy et al. [10] of around 190 nm. It was larger than that of bovine milk (~160 nm) [14], similar to that of ovine milk (~180 nm), and smaller than that of caprine milk determined using the same method (~210 nm) [19]. Heat treatment is known to increase the mean casein micelle size via both whey protein–casein micelle association and minor micelle–micelle aggregation [14,44]. Following the heat treatment of 95 °C for 5 min, the casein micelle size of the deer milk increased to 244 ± 8 nm (i.e., by about 25%). The extent of the heat-induced micelle size increase was similar to that of ovine milk (26%) and higher than those of caprine milk (16%) and bovine milk (6%) under comparable heating conditions [14,19]. This marked increase in micelle size in the deer milk can probably be attributed largely to micelle–micelle association during heat treatment, which would be promoted by the high protein content and ionic calcium concentration of the deer milk, as previously demonstrated in ovine milk [19,45].

The viscosity of the raw deer milk was 6.1 ± 0.6 mPa·s, higher than those of caprine milk (~3.8 mPa·s) and ovine milk (~4.6 mPa·s) [19]. The viscosity of milk increases naturally as the solids content increases, where fat content has a marked effect [46]. Pasteurization did not affect the viscosity of the deer milk. However, pasteurization following homogenization (250/50 bar) increased the viscosity of the deer milk markedly to 94 ± 67 mPa·s, which was further increased to 622 ± 437 mPa·s under the more intense heating condition of 95 °C for 5 min. There was a marked variation in the viscosity of the two homogenized milks. It appears that deer milk is sensitive to processing-induced instability (e.g., homogenization-induced clustering, heat-induced protein aggregation), probably arising from its high protein and fat contents, high ionic calcium concentration, large casein micelle size, and considerable heat-induced increase in micelle size (Figure 1 and Table 4). In addition, we determined the impact of processing on the viscosity using deer milks sampled from week 11 to week 16, which were particularly high in fat content and fat-to-protein ratio (Figure 1). The colloidal stability of processed deer milk is under further investigation in our group.

### 3.3. Gelation Properties of Deer Milk

#### 3.3.1. Acid-Induced Gelation

Table 5 presents the acid gelation properties of the differently processed deer milks. Acid gelation of the raw deer milk occurred at around 110 min of acidification when the mean pH was 4.83. The final raw deer milk acid gel had a G’ of 177 ± 5 Pa and a tan δ of 0.317. The gelation pH was close to that of bovine milk and higher than those of caprine and ovine milks reported previously [10,17,47]. The gel rigidity G’ agreed with that reported by Roy et al. [10], and was higher than those of bovine, ovine, and caprine milks [17]. The considerably higher protein and total solids contents of the deer milk compared with those of the other species probably played a key role. Li et al. [17] reported that the G’ values of raw bovine, ovine, and caprine milk gels followed the order of their protein contents.

Pasteurization at 75 °C for 15 s decreased the acid gelation time markedly to 62.2 ± 1.2 min, while also significantly increasing the gelation pH and G′ and reducing tan δ. These effects were further enhanced when pasteurization was combined with homogenization, attaining a mean gelation time of 45.4 min and a mean G′ of 234 Pa. Consistent with the milks of other ruminant species, homogenization and pasteurization slightly improved the acid gelation properties of the deer milk [17]. Pasteurization induces some whey protein denaturation, which promotes acid milk gelation, whereas homogenization allows better incorporation of large native milk fat globules into the gel matrix by breaking them into small fat globules covered by milk proteins. However, the effect of pasteurization alone on the acid gelation properties of the deer milk was greater than its effects on bovine, ovine, and caprine milks reported previously [17,48], particularly in terms of reducing the acid gelation time. A potential contributor may have been the higher protein content of the deer milk, which promotes greater whey protein denaturation and protein–protein interactions during heat treatment, and could subsequently contribute more to the acid gelation properties.

#### 3.3.2. Rennet-induced Gelation

Table 6 presents the rennet gelation properties of the deer milk. Raw deer milk had a fairly long rennet gelation time of 18.3 min, and the final rennet gel had a G′ of 384 ± 13 Pa and a tan δ of 0.30. When compared with raw bovine, ovine, and caprine milks studied using the same method [17,49], the rennet gelation time of the raw deer milk was similar to that of raw bovine milk and longer than those of raw caprine and ovine milks. It has been suggested that ovine and caprine casein micelles have lower colloidal stabilities, and thus require less cleavage of the κ-casein to induce coagulation [25,49]. The higher ethanol stability of the deer milk (Table 4) indicates greater colloidal stability of the casein micelles compared with caprine and ovine milks [19], which may have contributed to the longer rennet gelation time of the deer milk. In addition, given the higher protein content in the deer milk, a longer time would be required to reach the critical level of κ-casein cleavage in a sufficient number of casein micelles to induce gelation. The G′ of the rennet gels made from raw deer milk was higher than those of the rennet gels made from raw bovine, ovine, and caprine milks, which was naturally contributed by the high solids content of the deer milk. The tan δ of the raw deer milk rennet gel was similar to that of raw bovine milk gels, and lower than those of raw ovine and caprine milk gels, of around 0.4 [17]. The lower tan δ levels indicated that the deer milk rennet gel had more solid-like properties, with a smaller pore size and a lower tendency for syneresis compared with ovine and caprine milk rennet gels.

Both pasteurization and homogenization further improved the rennet gelation properties of the deer milk, reducing the gelation time and tan δ and increasing the final G′ of the gels. Similar effects of processing in decreasing tan δ have been reported for the other ruminant species [17]. However, only bovine milk, and not ovine or caprine milk, had a shorter rennet gelation time and a higher rennet gel rigidity following homogenization and pasteurization [17,50]. Overall, both the rennet gelation properties of the raw red deer milk and their processing-induced effects resembled those of bovine milk.

## 4. Conclusions

In this study, we characterized a wide range of compositional traits, structural characteristics, and gelation properties of New Zealand red deer milk. Deer milk is naturally nutritionally dense, being considerably richer in protein, fat, calcium, and zinc than other ruminant milks. Unique features of the deer milk fatty acids include their higher levels of C4:0, C18:0, α-linolenic acid, and BCFAs compared with other ruminant milks. The patterns of variation of individual minerals, fatty acids, and proteins in deer milk over a full lactation were identified, many of which differed from those demonstrated in bovine milk, suggesting the likely unique physiological and metabolic developments of lactating does. For other physicochemical properties, deer milk has large fat globules and casein micelles, as well as a fairly high ionic calcium concentration and viscosity. The impacts of processing on these characteristics were demonstrated. The acid- and rennet-induced gelation properties of deer milk were largely in line with those reported for other ruminant species, but, in many aspects, resembled those of bovine milk more than those of ovine milk or caprine milk. This study provides broad information on the production, composition, and techno-functional properties of deer milk. It will help in future developments of novel food and nutritional products utilizing red deer milk.

## Figures and Tables

**Table 1:** Mineral composition of deer milk and its variation over the lactation.

Mineral	Mean ± SD	Minimum–Maximum	Correlation Coefficient with Lactation Weeks ^1^
Calcium (mg/100g)	297.5 ± 18.3	260–310	−0.30
Magnesium (mg/100g)	16.8 ± 1.0	14.9–17.7	−0.22
Potassium (mg/100g)	135.6 ± 11.1	113–151	−0.85 **
Sodium (mg/100g)	38.0 ± 4.4	33–45	0.90 **
Chloride (mg/100g)	76.8 ± 11.3	66–97	0.89 **
Phosphorus (mg/100g)	208.1 ± 13.6	185–230	−0.66
Zinc (mg/kg)	9.56 ± 1.16	8.30–12.0	−0.56
Iodine (mg/kg)	0.69 ± 0.37	0.34–1.44	−0.09
Copper (mg/kg)	0.16 ± 0.15	0.05–0.48	−0.88 **
Selenium (mg/kg)	0.02 ± 0.01	0.01–0.04	0.67

^1^ Significant correlations are indicated: *p* < 0.01 **.

**Table 2:** Fatty acid composition (g fatty acid/100 g) of deer milk.

Fatty Acid	Mean ± SD	Minimum–Maximum	Lactation Effect ^1^
C4:0	5.69 ± 0.18	5.31–5.92	NS
C6:0	2.24 ± 0.30	1.93–2.66	Decrease
C8:0	1.32 ± 0.29	1.03–1.75	Decrease
C10:0	2.25 ± 0.52	1.72–3.01	Decrease
C10:1	0.14 ± 0.02	0.12–0.16	Decrease
C12:0	2.85 ± 0.60	2.26–3.72	Decrease
iso C14	0.15 ± 0.02	0.13–0.19	Quadratic
C14:0	12.34 ± 1.46	10.53–14.86	Decrease
iso C15	0.47 ± 0.07	0.37–0.57	Quadratic
anteiso C15	0.66 ± 0.11	0.44–0.79	Quadratic
C14:1	0.24 ± 0.16	0.16–0.62	NS (Highest in week 3)
C15:0	1.08 ± 0.11	0.90–1.17	NS (Quadratic *p* = 0.08)
iso C16	0.29 ± 0.05	0.23–0.37	Quadratic
C16:0	25.81 ± 1.15	24.59–27.86	NS
iso C17	0.82 ± 0.05	0.75–0.90	NS
C16:1	0.66 ± 0.62	0.36–2.16	NS (Highest in week 3)
anteiso C17	0.46 ± 0.06	0.35–0.56	Quadratic
C17:0	0.68 ± 0.08	0.54–0.79	NS (Quadratic *p* = 0.07)
C17:1	0.08 ± 0.01	0.07–0.10	NS
C18:0	16.83 ± 2.18	12.34–18.83	Increase
C18:1 t9	0.16 ± 0.02	0.13–0.18	NS
C18:1 t11	1.72 ± 0.25	1.25–2.00	Cubic
C18:1 c9	15.11 ± 1.37	13.09–17.09	Increase
C18:1 c11	0.78 ± 0.31	0.60–1.54	NS (Highest in week 3)
C18:2 n6	1.07 ± 0.16	0.87–1.31	NS
C20:0	0.25 ± 0.05	0.13–0.29	Increase
C18:3 n3	1.48 ± 0.38	1.08–2.25	Cubic
CLA	0.45 ± 0.11	0.30–0.58	Cubic
C22:5	0.20 ± 0.03	0.17–0.25	Cubic
C22:6	0.07 ± 0.04	0.03–0.13	Cubic
C14:1/C14:0	0.019 ± 0.011	0.013–0.046	NS (Highest in week 3)
C16:1/C16:0	0.026 ± 0.024	0.014–0.084	NS (Highest in week 3)
C18:1 c9/C18:0	0.908 ± 0.118	0.784–1.182	NS (Highest in week 3)
CLA/C18:1 t11	0.261 ± 0.035	0.203–0.299	Cubic

^1^ Lactation effect: this table presents the regression results of the fatty acid data fitting to linear, quadratic, and cubic regression models at a significance level of *p* < 0.05. SD, standard deviation; NS, nonsignificant; CLA, cis-9,trans-11 conjugated linoleic acid.

**Table 3:** Relative protein composition of deer milk (presented as percentages of HPLC peak area of individual proteins in all identified proteins).

Protein Peak	Mean ± SD	Minimum–Maximum	Correlation Coefficient with Lactation Weeks ^1^
κ-Casein	5.2 ± 0.9	3.9–6.5	0.96 ***
α_s2_-Casein	5.8 ± 1.1	4.8–7.9	−0.87 **
α_s1_-Casein	2.4 ± 0.3	1.8–2.7	−0.32
β-Casein	49.7 ± 0.7	48.6–50.8	0.12
Unknown	20.7 ± 2.3	15.9–22.8	0.81 *
Whey protein 1	4.7 ± 0.7	4.1–6.2	−0.90 **
Whey protein 2	9.8 ± 0.7	9.1–11.1	−0.86 **
Whey protein 3	1.8 ± 0.5	1.2–2.6	−0.98 ***

^1^ Significant correlations are indicated at different levels: *p* < 0.05 *; *p* < 0.01 **; *p* < 0.001 ***.

**Table 4:** Physicochemical properties of differently processed deer milks *.

Property	RM	PM	HPM	HHM
pH	6.67	6.67	6.67	6.67
Ionic calcium concentration (mM)	3.34 ^a^	3.10 ^b^	3.08 ^b^	3.07 ^b^
Ethanol stability (%)	59.4 ± 1.3	ND	ND	ND
Fat globule size (μm, *D* [4,3])	6.66 ± 0.10	ND	ND	ND
Casein micelle size (nm)	195 ± 4 ^b^	199 ± 5 ^b^	ND	244 ± 8 ^a^
Viscosity (mPa·s)	6.1 ± 0.6	6.0 ± 0.5	94 ± 67	622 ± 437

* RM, raw milk; PM, pasteurized milk (75 °C for 15 s); HPM, homogenized and pasteurized milk (200/50 bar; 75 °C for 15 s); HHM, homogenized and heated milk (95 °C for 5 min); *D* [4,3], volume-weighted mean (μm); ND, not determined. ^a,b^ Means with different superscripts within the same row differ significantly (*p* < 0.05, one-way ANOVA with the Tukey post hoc test).

**Table 5:** Acid gelation properties of differently processed deer milks *.

Property	RM	PM	HPM
Acid gelation time (min)	113.4 ± 8.2 ^a^	62.2 ± 1.2 ^b^	45.4 ± 6.7 ^c^
Gelation pH	4.83 ± 0.02 ^c^	5.05 ± 0.02 ^b^	5.14 ± 0.04 ^a^
Storage modulus G’ (Pa)	177 ± 5 ^c^	208 ± 3 ^b^	234 ± 11 ^a^
Tan δ	0.317 ± 0.003 ^a^	0.276 ± 0.004 ^b^	0.259 ± 0.012 ^b^

* RM, raw milk; PM, pasteurized milk (75 °C for 15 s) milk; HPM, homogenized and pasteurized milk (200/50 bar; 75 °C for 15 s). ^a,b,c^ Means with different superscripts within the same row differ significantly (*p* < 0.05, one-way ANOVA with the Tukey post hoc test).

**Table 6:** Rennet gelation properties of differently processed deer milks *.

Property	RM	PM	HPM
Rennet gelation time (min)	18.3 ± 2.1 ^a^	16.1 ± 1.2 ^ab^	14.5 ± 0.8 ^b^
Storage modulus G’ (Pa)	384 ± 13 ^c^	457 ± 22 ^b^	529 ± 39 ^a^
Tan δ	0.298 ± 0.004 ^a^	0.281 ± 0.010 ^b^	0.263 ± 0.0051 ^c^

* RM, raw milk; PM, pasteurized milk (75 °C for 15 s); HPM, homogenized and pasteurized milk (200/50 bar; 75 °C for 15 s). ^a,b,c^ Means with different superscripts within the same row differ significantly (*p* < 0.05, one-way ANOVA with the Tukey post hoc test).

## Data Availability

Data generated during the study are available from the corresponding authors upon request.
